# Hepatitis C Transmission and Treatment in Contact Networks of People Who Inject Drugs

**DOI:** 10.1371/journal.pone.0078286

**Published:** 2013-11-01

**Authors:** David A. Rolls, Rachel Sacks-Davis, Rebecca Jenkinson, Emma McBryde, Philippa Pattison, Garry Robins, Margaret Hellard

**Affiliations:** 1 Melbourne School of Psychological Sciences, University of Melbourne, Melbourne, Victoria, Australia; 2 Centre for Population Health, Burnet Institute, Melbourne, Victoria, Australia; 3 Department of Epidemiology and Preventive Medicine, Monash University, Melbourne, Victoria, Australia; 4 Centre for Research Excellence in Injecting Drug Use, Burnet Institute, Melbourne, Victoria, Australia; 5 Department of Medicine-RMH, University of Melbourne, Victoria, Australia; University of California, United States of America

## Abstract

Hepatitis C virus (HCV) chronically infects over 180 million people worldwide, with over 350,000 estimated deaths attributed yearly to HCV-related liver diseases. It disproportionally affects people who inject drugs (PWID). Currently there is no preventative vaccine and interventions feature long treatment durations with severe side-effects. Upcoming treatments will improve this situation, making possible large-scale treatment interventions. How these strategies should target HCV-infected PWID remains an important unanswered question. Previous models of HCV have lacked empirically grounded contact models of PWID. Here we report results on HCV transmission and treatment using simulated contact networks generated from an empirically grounded network model using recently developed statistical approaches in social network analysis. Our HCV transmission model is a detailed, stochastic, individual-based model including spontaneously clearing nodes. On transmission we investigate the role of number of contacts and injecting frequency on time to primary infection and the role of spontaneously clearing nodes on incidence rates. On treatment we investigate the effect of nine network-based treatment strategies on chronic prevalence and incidence rates of primary infection and re-infection. Both numbers of contacts and injecting frequency play key roles in reducing time to primary infection. The change from “less-” to “more-frequent” injector is roughly similar to having one additional network contact. Nodes that spontaneously clear their HCV infection have a local effect on infection risk and the total number of such nodes (but not their locations) has a network wide effect on the incidence of both primary and re-infection with HCV. Re-infection plays a large role in the effectiveness of treatment interventions. Strategies that choose PWID and treat all their contacts (analogous to ring vaccination) are most effective in reducing the incidence rates of re-infection and combined infection. A strategy targeting infected PWID with the most contacts (analogous to targeted vaccination) is the least effective.

## Introduction

Hepatitis C virus (HCV) is a blood-borne virus which chronically infects over 180 million people worldwide [1], and disproportionately affects people who injects drugs (PWID). The sharing of needles, syringes and ancillary equipment is believed to be the primary means of transmission, accounting for the majority of new infections [2]–[4] (∼90% in Australia, ∼72% in Canada, and ∼54% in the United States). HCV has significant morbidity and mortality with an estimated 350,000 deaths annually attributed to HCV-related diseases including cirrhosis and hepatocellular carcinoma [1]. In the United States more deaths are now attributed to HCV than HIV [5]. Unlike for hepatitis A or B, currently there is no preventative vaccine for HCV.

Current treatment for HCV generally ranges from 24–48 weeks of pegylated interferon and ribavirin depending on the HCV genotype, IL28B genotype and stage of hepatic fibrosis. Increasingly, HCV treatment is becoming “response based” with the length of treatment varying based on how quickly a patient’s viral load becomes undetectable. Current treatments are estimated to be effective in about 60% [6]–[8] of cases, again varying depending on HCV genotype, IL28B genotype and level of hepatic fibrosis. Treatment rates of infected PWID remain low for a combination of reasons including lack of awareness by PWID of their infected status, reluctance by some PWID to undergo treatment due to significant treatment side effects, reluctance by some clinicians and health services to treat PWID due to concerns about low levels of treatment success despite increasing evidence that this is not the case [9], and concern about high levels of HCV re-infection in PWID despite limited evidence that this occurs [10]–[12].

Over the next five years there will be major changes in HCV treatment. With the advent of direct-acting antiviral medications, treatment will become more efficacious, of shorter duration and will have less severe side effects. As well as benefiting individual patients, recent mathematical modelling suggests that treating PWID can lead to a considerable reduction in HCV prevalence over time due to a decrease in HCV transmission within the PWID community [13], [14].

Previous models of HCV transmission have typically made some assumption of “mixing” rather than consider the contact network of PWID (e.g. [15]–[22], and [2], [14], [23], [24] in the Australian context) or have lacked an empirically grounded contact network [25]. Under a homogeneous mixing assumption, members of a population are assumed to have contact with all other members of the population [26]. It is increasingly recognised that contact networks are relevant to the transmission of disease [27], [28], especially close-contact diseases [29]. In the context of HCV and the “hidden population” of PWID, data collection [30]–[35] using network-based methods is largely in its infancy. Network-based modelling efforts into HCV and PWID contact networks are even newer. Recently Rolls et al. [36] developed a transmission model for HCV in conjunction with an empirical snowball sampled network of PWID and an empirically grounded contact network model of PWID [37], while Khan et al. [38] have produced a contact network model of PWID using data from the Social Factors and HIV Risk study [39], [40]. (Limitations of the network model in [38] are discussed in [37].) To date no modelling of HCV transmission and treatment with empirically grounded contact networks has been done.

Most of the research into network-based interventions to limit disease transmission has involved network contact modification such as isolation (e.g., for SARS [41]) or vaccination rather than treatment (e.g., for HCV [21]). Some network-based vaccination strategies require knowledge about the entire network whereas others only require information local to individuals. *Targeted vaccination* (e.g., [42], [43]) involves targeting nodes in decreasing order of number of contacts, which requires knowing the number of contacts of all members of the network. Other measures such as betweenness or closeness centrality, instead of number of contacts, have also been considered (e.g., [44]). In contrast, *ring vaccination* targets all the primary contacts of target cases (as for hepatitis B [45]), or primary contacts and secondary contacts (i.e., contacts of primary contacts) as for smallpox eradication [46], [47]. *Acquaintance vaccination* and its variants [43], [48], [49] target primary contacts whose number of contacts are above some predetermined value.

In practice, the entire network is usually unknown, so strategies requiring local information are most clinically relevant. Research into these strategies usually assumes the contact network has rare nodes with very large numbers of contacts (e.g., “scale-free” networks [50]). Such highly connected nodes are sometimes called “hubs”. For such a network, compared to vaccinating randomly chosen nodes, acquaintance vaccination strategies have been demonstrated to be more effective in reducing outbreak size (e.g., [42], [43], [48], [49]). For a contact network without such hubs, the difference in strategies appears much smaller (e.g. [42], [44], [51]) although the modelling study by Hartvigsen et al. [52] showed targeted vaccination against influenza reduced outbreak size somewhat better than random node selection in simulated networks without hubs. In the context of HCV treatment, lack of hubs in the contact network would mean strategies based on finding and removing hubs as a source of infection will probably not be particularly advantageous. Moreover, the possibility of re-infection after treatment means network modelling results that assume immunity is possible may not apply to HCV.

The study by Porco et al. [47] is notable for considering ring vaccination including secondary contacts for a smallpox outbreak on a network without hubs. Instead they use a network model capturing household structure, in which each individual is a member of both a fully-connected “household” of mean size four and a fully-connected non-household workplace/social group of mean size eight. Probability of transmission is assumed to be higher in the household group. They find ring vaccination can be a successful strategy for halting a smallpox epidemic, but do not compare with other strategies, or use empirically grounded networks. Furthermore, in contrast to our study, they study vaccination rather than treatment, after which there is no possibility of re-infection.

Simulation models provide an effective method to investigate disease transmission and to conduct controlled experiments to explore the potential benefits of possible treatment strategies. Here we explore HCV transmission and possible treatment strategies on empirically grounded simulated PWID contact networks. Our work builds on our previous efforts creating both an individual-based transmission model [36] and an empirically grounded contact network model of PWID [37] using data collected in Melbourne, Australia, in a study that used network methods [34], [35]. Using molecular epidemiological techniques, it has recently been demonstrated that clusters of related HCV infection in the Melbourne study cohort are correlated with network distance in the snowball sampled empirical contact network [53], justifying the use of the empirical contact network as a basis for studying HCV transmission.

Our network model [37] is from the class of exponential random graph models (ERGMs) [54]–[56]. ERGMs are a class of probabilistic network models grounded in hypotheses about social processes underlying network formation, and are commonly used in social network analysis. ERGMs capture network features and structures relevant to human interaction such as *transitive closure*, *homophily* and *social circuit dependence*. Transitive closure, sometimes called *clustering*, is a key feature of social networks, and refers to the propensity for triangles to form. It is typified by the adage “the friend of my friend is also my friend”. Homophily is the tendency to form contacts with others that share similar attributes (e.g., age, gender). It is typified by the adage “birds of a feather flock together”. Loosely, social circuit dependence captures the idea that people whose contacts are connected are themselves more likely to be connected. Recent advances with new ERGM specifications [57], [58] provide sophisticated methodology such that empirical networks with these features can often be modelled parsimoniously.

This work studies HCV transmission and treatment in the context of empirically grounded contact networks. In the context of treatment we investigate an anticipated HCV treatment rather than preventative vaccines, starting in a situation where HCV is essentially endemic, infecting about half the network. We directly compare a number of network-based interventions in this population, including ring vaccination with secondary contacts. In the context of transmission we investigate the role of the number of contacts and injecting frequency on time to primary infection and the role of spontaneously clearing nodes on incidence rates. Importantly, in this study the PWID contact network model is empirically grounded and the transmission model includes “imported infections” which recognise both the limitations around including all network partners in empirical studies and the limitations of using a static network to model time intervals longer than those used to define a contact.

## Methods

### Transmission Model

Details of our transmission model have appeared elsewhere [36]. In short, it is a stochastic individual-based model which simulates HCV transmission within a static network on a week-by-week basis. Any node in the network can be infected by an infected network neighbour according to a yes/no probability of sharing followed by a yes/no probability (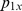) of transmission from a sharing event. Probabilities of sharing depend on the injecting frequency of the two nodes (each either less than daily or at least daily). In addition to infection by network transmission, infections can also be “imported” meaning the source of infection is not a network neighbour. Imported infections provide a way to include risks from under-reporting of network neighbours and small changes to the network which would otherwise not be included using a static network model. Most parameter values are based on values published in the literature. Exceptions to this are the sharing probabilities and mean incidence rate of imported infection which are estimated from sharing and infection data collected in the Melbourne study [34]–[36], and the probability of transmission from a sharing event (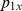) which was found by calibration using infection data and an empirical network from the Melbourne study [36].

A feature of our model is that a fraction (25% [59] unless otherwise mentioned) of infected nodes can clear spontaneously in the acute phase. No acquired immunity is assumed in spontaneously clearing nodes so these nodes can and do cycle between being susceptible and infected. The ability to spontaneously clear is assigned to nodes randomly prior to simulating transmission, independent of other features. The set of spontaneously clearing nodes varies from one simulation to the next. This is further clarified below in connection with “burn-in”. For such nodes, the duration of each infection is simulated from an exponential distribution, independent of other durations.

Model calibration for 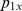 is based on an RNA prevalence of 56% (i.e., 56% of the network is infected.) Thus, simulations include a burn-in phase in which 30% of the nodes are initially infected and the simulation proceeds until the prevalence reaches 56% (on average across 200 simulations.) One key difference from the model described in [36] is that the incidence rate of imported infection is now allowed to vary according to the prevalence at the end of the previous week. This recognises that a community-based treatment strategy would typically also lower the prevalence beyond the network that we have modelled, and so the rate of imported infections should be reduced. The mean incidence rate of imported infection (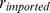) is related to the prevalence (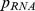) through the equation
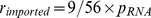
(1)where 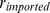 is measured in person-years (PY) at risk. Notice this is a linear relationship for which there are no infections if the prevalence is zero, and the mean rate is 9 per 100 person-years at risk when the prevalence is 0.56, which agrees with the calibration in [36]. (The use of a linear relationship can also be supported as a reasonable approximation, for example, if the numbers of unseen network partners for a network node have a Poisson distribution. The result is not shown here for brevity.)

### Network Model

Details of our contact network model have been described elsewhere [37]. In short, the Melbourne study was a network based data collection from three urban locales in the Melbourne area from which an empirical contact network was created. Using molecular epidemiogical techniques [53], correlation between distances in the empirical contact network and clusters of related HCV infection have been demonstrated, providing re-assurance that the empirical contact network is the right network to look at. For various reasons [36], [37], in order to model the transmission network a contact was defined as two people participating in injecting behaviour in the same room or place and roughly the same time, as opposed to a narrower definition requiring a participant to report actual sharing of a syringe, in the previous three months. (There was no study question about sharing of ancillary equipment.) In this sense, the empirical network created is a network of opportunity for HCV transmission.

Using this empirical network and results from social network analysis [57], [60], [61], an ERGM was fit to the data [37]. Table 1 shows the model specification. It is a model for the contact network in the street drug scene in three suburbs of Melbourne, Australia. Specifically it models connected components with at least three people (so no isolates or isolated pairs). The size of the network was estimated to be about 524 people. The model includes five parameters for network structure: edge (for controlling edge density), isolates (for keeping the number of isolates near zero), alternating-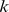-star (which is useful for modelling the node degree distribution), and alternating-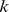-triangle and alternating-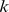-2-path (useful for modelling both clustering and social circuit dependence). In addition, four parameters model homophily effects: location (1, 2, 3), gender (M/F), age (less than 25, greater than 25), and injecting frequency (less than daily, at least daily). A positive homophily parameter indicates a propensity for two PWID to share a network tie when they have that attribute in common. In this ERGM all four homophily parameters are positive, although homophily on gender was included for completeness but not found to be a significant effect [36]. With this model we can simulate empirically grounded contact networks with which to simulate HCV transmission.

**Table 1:** ERGM specification for PWID contact network.

Parameter	Parameter Value
Edge	−8.384
Isolates	−9.308
Alternating-*k*-star	0.611
Alternating-*k*-triangle	1.707
Alternating-*k*-2-path	−0.563
Same location	2.111
Same gender	0.280
Same age<25	0.787
Same daily user freq.	0.429

Specification for the PWID contact network ERGM. The first five parameters model network structure while the last four model homophily effects: location (1, 2, 3), gender (M/F), age (less than 25, greater than 25), and injecting frequency (less than daily, at least daily). Positive homophily parameters indicate a propensity for two PWID to share a network tie when they have that attribute in common.

**Figure 1:**
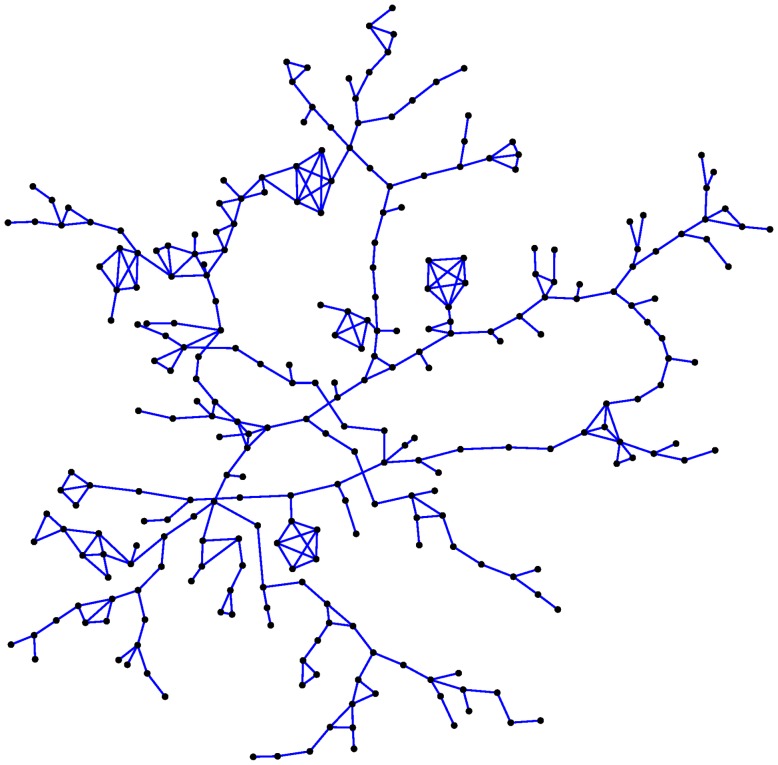
Typical simulated PWID network with 274 nodes.

**Figure 2:**
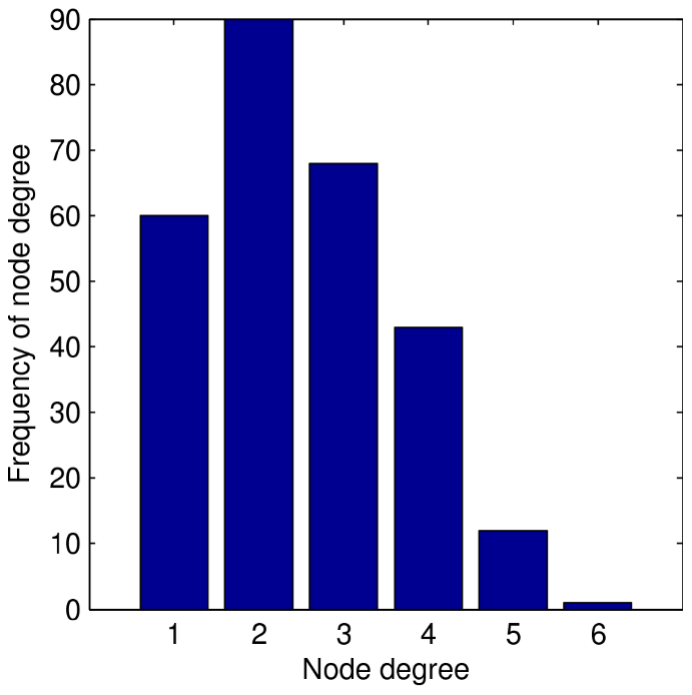
Histogram of node degree for the network shown in Figure 1.

For the results reported here we use 100 simulated networks, each of which has 274 nodes. We form these networks by using the ERGM to simulate many networks with 524 nodes, and keep the first 100 largest components that have 274 nodes. The size 274 was chosen simply because it was the mode of the distribution of largest component sizes across 48,000 simulated networks reported previously [37]. We expect similar results for other big components, although using a consistent component size provides the most controlled comparisons. We focus on the largest component for the obvious reason that the role of the network is more interesting for investigation than in isolated pairs and triples. In addition, there are modelling challenges around the number of isolated nodes, pairs and triples in the community because they are harder to find in a study, and there may even be a separate network mechanism by which some people actively try to stay in a small contact network. Such issues are beyond the scope of our network model, so we focus on the largest component only. Figure 1 shows a typical network used in this study. Figure 2 shows the histogram for the number of contacts, or “node degree”, of the same network. In particular, notice these networks do not have “hubs” (i.e., the node degree distribution does not have extreme outliers characteristic of a “fat tailed” distribution). While we deal with a single component in isolation, a community model could easily be imagined as a collection of such components. Since results reported here are averages across 100 different components, combining several such components in a population model would not change our conclusions.

### Model Scenarios

We conducted three sets of simulations. The first set was designed to investigate the role of network features on the time to primary infection in the baseline transmission model. (Throughout, an infection is “primary” if the node was never previously infected, including in the burn-in phase. Otherwise, an infection is counted as a “re-infection”.) Key parameter values are listed in Table 2. Other values are the same as previously described [36]. No community treatment strategies were included. To investigate time to primary infection, 3,000 simulations were performed for 520 weeks beyond the end of burn-in for each of 100 networks. Each simulation used a different burn-in with its own collection of randomly assigned seed nodes and spontaneously clearing nodes. For nodes never infected during the burn-in, the time to primary infection was recorded if infected during the simulation. If the node did not become infected, the censored value of 520 was recorded.

**Table 2:** Key Model Parameters for Simulations.

Model Parameter Definition	Value	Reference
Prob. of transmission from one sharing event ( 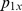 )	0.00995	[36]
Rate of importing infection into a node ( 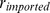 )	varies	[36]
Proportion of spontaneously clearing nodes	0.25	[59], [79]
Prevalence at end of burn-in phase	0.56	[36]
Edgewise weekly probability of sharing (both less-frequent users)	0.19	[36]
Edgewise weekly probability of sharing (one less-frequent user)	0.18	[36]
Edgewise weekly probability of sharing (two more-frequent users)	0.24	[36]
Incidence rate ratio for imported infections of freq. vs. non-freq. users	1.3	[36]
Mean time to chronic spontaneous clearance (years)	200	[80]
Duration of latent period (weeks)	2	[81]
Mean time to acute spontaneous clearance (weeks)	7	[79]
Duration of acute phase (weeks)	26	by definition

Key model parameters used for transmission and treatment simulations. Less-frequent users have injecting behaviour less than weekly (on average) while more-frequent users have injecting behaviour at least weekly (on average). Rate of importing infection [36] is modified to account for varying prevalence.

The second set of simulations was designed to investigate the impact of network features (e.g., arrangement of spontaneously clearing nodes, number of spontaneously clearing nodes) on the incidence rate of total infection (i.e., primary or re-infection) in the baseline transmission model. Again, no community treatment strategies were included. By creating sets of nested simulations and fixing certain features (e.g., spontaneously clearing group, number of spontaneously clearing nodes, etc.) we can isolate their effect on the incidence rate of total infection. First, groups of spontaneously clearing nodes were created (referred to as S1, S2, 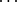.) Since spontaneous clearing is assigned randomly, the number and location of these nodes within the network varies between the groups. For each group of spontaneously clearing nodes, 15 burn-ins were simulated each. (So, for the spontaneously clearing group S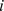 the burn-ins are S
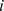
B1, S
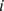
B2, 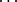, S
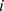
B15 and so on.) Across burn-ins the location of initially infected seed nodes varies. Finally, for each burn-in, 15 simulations were performed for 52 weeks after burn-in. (So for burn-in S
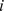
B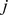 the simulations are S
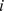
B
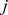
sim1, S
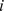
B
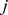
sim2, 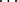, S
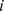
B
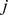
sim15.) The duration is long enough to perform investigations while being conservative to the possibility that the network changes over time.

The third set of simulations was designed to investigate the effect of treatment strategies on both the incidence rate of infection and prevalence. For each of the nine treatment strategies, 500 simulations were performed (a different burn-in for each) for each of seven treatment initiation frequencies (i.e, treatment “epochs” to find and begin treating new people are regularly spaced every 1, 2, 4, 8, 13, 26 or 52 weeks.) This is equivalent to treatment coverage varying from 3.7–190 treatment initiations per 1000 PWID per year if each epoch corresponds to one treatment initiation. These simulations cover a period of 156 weeks (i.e., three years) following burn-in which provides enough time that differences between the strategies emerge. We made the following assumptions about treatments based on projected characteristics of direct-acting antivirals that are currently under development [62]. Treatment is effective in 80% of people. Only infected people are treated, and they will not start a new course of treatment if in the middle of a course of treatment. The duration of treatment is 12 weeks. If treatment is effective the duration of infectiousness was conservatively estimated to be 10 weeks and if treatment is not effective they remain infectious throughout treatment. Those who fail to obtain a sustained virological response (treatment success) are not eligible to be retreated. Thus, nodes “available” for treatment are those infected nodes not currently in treatment without a history of previous treatment failure. For strategies that treat network contacts as well, all referred contacts begin treatment in the same week as the person who referred them (whom we call “ego” in reference to the social network literature).

For these simulations, different burn-ins have different random collections of spontaneously clearing nodes, different random collections of nodes for whom treatment is effective, and different random arrangements of infected nodes at the end of burn-in. By averaging over the 500 simulations the differences between strategies can be separated from random “noise”. Averaging over the 100 networks has a similar effect on the random selection of networks. To further minimise the effects of random noise, the post-burn-in simulations were also organized as a series of controlled experiments, where the control group was the baseline simulations using the results from the 50 000 burn-ins (500 per network, 100 networks) as initial configurations. Simulations for each of the nine treatment strategies used the same 50 000 burn-ins as initial configurations. In total, the investigation of treatment strategies involved over 3 million post-burn-in simulations (500 simulations×9 strategies×7 frequencies×100 networks).

We consider nine treatment strategies in all. One strategy uses no network information, two strategies use “global” information about the network, and six use information local to individual nodes. We further describe these strategies here. They are summarised in Table 3.

**Table 3:** Treatment Strategies.

Strategy	Short Name	Node Selection at each Treatment Epoch
Decreasing node degree	dec. degree	Choose node avail. for treatment with largest node degree.
Increasing node degree	inc. degree	Choose node avail. for treatment with smallest node degree.
Random node selection	random	Choose avail. ego randomly. Treat ego.
Acquaintance, degree ≥5	acq5	Choose avail. ego randomly. Treat ego & ego’s avail. contacts with node degree ≥5.
Acquaintance, degree ≥3	acq3	Choose avail. ego randomly. Treat ego & ego’s avail. contacts with node degree ≥3.
Primary contacts	ring	Choose avail. ego randomly. Treat ego & ego’s avail. contacts.
Primary & some sec. contacts	2-ring	Choose avail. ego randomly. Treat ego, avail. prim. contacts and some avail. sec. contacts.
Primary and all sec. contacts	2-ring all	Choose avail. ego randomly. Treat ego, avail. prim. contacts, and all avail. sec. contacts.
Contacts of uninfected nodes	naive ring	Choose uninfected ego randomly. Treat all of ego’s avail. prim. contacts.

Abbreviations: “avail.”: available, “prim.”: primary, “sec.”: secondary.

Treatment strategies considered. In all cases, only infected nodes not currently in treatment and without a history of treatment failure are “available” for treatment.

#### 1. Treatment strategy: random node selection

The treatment strategy (“random”) selects a node at random at each treatment epoch from the collection of available nodes. Thus, no network information is used. For this strategy there is a clear, non-random relationship between the treatment frequency and the mean number of treatment starts per 1000 PWIDs. For example, new treatment epochs every fourth week would see 13 people treated per year or about 47 people yearly per 1000 in a network component of size 274.

#### 2. Treatment strategies: priority by node degree

We consider two treatment strategies that use “global” information about the network. That is, at each treatment epoch, the strategies rank the available nodes in priority order for treatment, either by order of increasing (“inc. degree”) or decreasing (“dec. degree”) node degree and choose the highest ranked node for treatment. Taking nodes in decreasing order is analogous to targeted vaccination. Since knowing all node degrees and knowing the current infection status of all nodes in the PWID network will both generally be impossible, these are not practical strategies. However, they can serve as useful benchmarks. Indeed, amongst vaccination strategies the best known strategy on scale-free networks is believed to be targeted vaccination [43]. As with random node selection, there is a clear, non-random relationship between the treatment frequency and the mean number of treatment starts per 1000 PWIDs.

#### 3. Treatment strategies: primary contacts

By analogy with ring vaccination, for ring treatment (“ring”), at each treatment epoch one node (“ego”) is chosen at random from those available for treatment, and treated. In addition, all of ego’s primary contacts (i.e., ego’s “ring”) which are available for treatment, are treated. Across simulations the number of treatment initiations will vary depending on node degrees and the number of neighbours actually infected.

We also consider two treatment strategies, analogous to enhanced acquaintance immununization, in which we treat ego, chosen at random, and certain members of ego’s ring that are available for treatment. The criteria for their treatment is that their number of contacts (i.e., node degree) is at least some cutoff: either 5 (“acq5”) or 3 (“acq3”). Note that unlike enhanced acquaintance immununization, we also treat ego. Also note that “acq
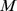
” (where 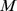 is larger than the maximum node degree) would correspond to random node treatment while acq0 would correspond to ring treatment (in the absence of isolated nodes.).

#### 4. Treatment strategies: primary and secondary contacts

We consider two treatment strategies that include primary and secondary contacts. There are two strategies because a secondary contact could be defined as all the additional neighbours of *all* of ego’s ring (“2-ring all”), or just the additional neighbours of the *infected* members of ego’s ring (“2-ring”). As with other strategies, only those available for treatment are treated. As with the ring strategy, across simulations the number of treatment starts will vary.

Thus the four strategies “random”, “acq5”, “acq3”, and “ring” capture a spectrum of strategies that begin with a randomly chosen ego at each treatment epoch and treat an increasing fraction of ego’s primary contacts, while the “2-ring” and “2-ring all” strategies go even further by treating an increasing fraction of ego’s secondary contacts too.

#### 5. Treatment strategy: primary contacts of uninfected nodes

Finally, we consider an additional treatment strategy (“naive ring”) which treats the infected primary contacts of randomly selected HCV-naive (i.e., never infected) nodes. This is the only strategy for which the randomly chosen node is not available for treatment. We caution that results for this strategy must be viewed as preliminary. Our network model does not explicitly model the contacts of new injectors. Thus, it assumes their contacts are similar to more experienced injectors, and so results for this strategy will be the most sensitive to departures from this assumption. We discuss this further in the Discussion.

### Analysis

Network visualisation was created using Pajek [63]. Simulations, analyses and boxplots were completed using MATLAB [64]. For incidence rates of infection, infections are counted from the start of the post-burn-in phase. Incidence rates are computed using the number of infections and weeks susceptible in the post-burn-in phase. Nested ANOVA analyses use the anovan function. Confidence intervals for mean incidence rates and mean proportions use a Gaussian approximation. Kaplan-Meier estimates were generated using the ecdf function.

## Results

### Transmission

**Figure 3:**
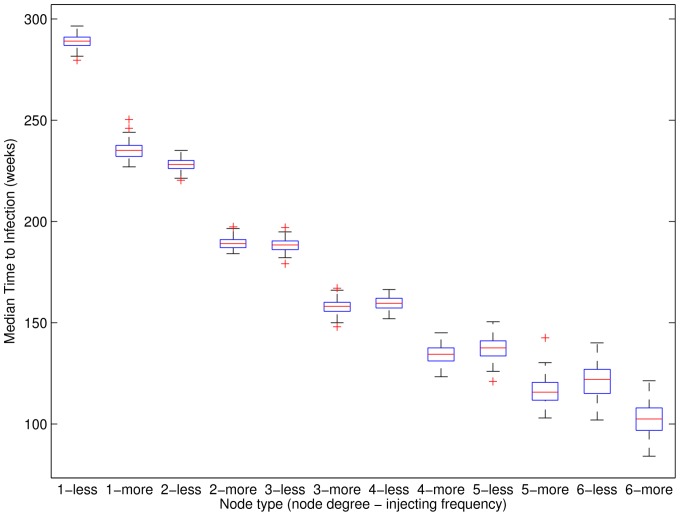
Median time to primary infection across 100 simulated networks. Boxplots are for results for each of 12 categories (node degrees 1–6; two injecting frequencies) over 100 networks. Injecting behaviour frequency is denoted as “less” (i.e., less than daily) or “more” (i.e., at least daily). For each network, results are formed from 3000 HCV simulations as the median for each node as both a less frequent and a more frequent injector, and then the median for each of the 12 groups. Boxes show the 25-th and 75-th percentiles. The central line denotes the median, the whiskers show the range of data not considered outliers, and outliers are shown individually. More frequent injecting behaviour is approximately equivalent to being a less frequent injector with one additional network contact.

As expected, both increased numbers of contacts (i.e., node degree) and increased injecting frequency play key roles in reducing the time to primary infection. Figure 3 shows median time to primary infection for node degrees 1–6 and both injecting frequencies as boxplots across 100 networks. Results for each network are calculated as the median for each node separately as a less frequent and a more frequent injector across 3000 HCV simulations. These are then combined by forming the median for each of the 12 categories for that network. Boxes show the 25-th and 75-th percentiles. The central line denotes the median, the whiskers show the range of data not considered outliers, and outliers are shown individually. Several results are clear. Time to primary infection is noticeably reduced for each additional sharing partner when the number of sharing partners is small (e.g. by about one year between degree 1 and 2). It is also clear that compared to a node injecting less than daily, the reduced time to primary infection for nodes injecting at least daily is roughly the same as the reduced time from having an additional sharing partner. Finally, the variation in the time to primary infection across the 100 random networks for fixed node degree and injecting frequency is small compared to the variation across node degrees and injecting frequencies. This shows that for our simulated ERGM networks, once network-wide prevalence and incidence rate are accounted for (i.e., by burn-in and calibration, respectively), node heterogeneity plays a larger role than network variation in determining a node’s time to primary infection.

We investigated the role of spontaneously clearing nodes on the incidence rate of total infection (i.e., primary or re-infection) using analysis of variance (ANOVA) and nested models in which the burn-in group is nested within the particular group of spontaneously clearing nodes. The number and locations of spontaneously clearing nodes varies randomly across the groups of spontaneously clearing nodes. For a single fixed network of size 274, simulation results from 15 randomly chosen groups of spontaneously clearing nodes (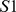, 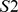, 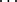, 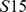) were investigated. The nested ANOVA results show the effect of the particular group of spontaneously clearing nodes on the incidence rate of total infection is statistically significant (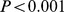). That is, there is a connection between the spontaneously clearing group and the incidence rate of total infection, either from their number, location or both. On the other hand, if 15 groups of spontaneously clearing nodes are chosen such that all have either 64 or 65 spontaneously clearing nodes (the two most common values), the spontaneously clearing group is no longer significant (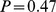). Since location is the only remaining feature of the spontaneously clearing nodes that can vary, this shows that the *locations* of spontaneously clearing nodes is not statistically significant for the network-wide incidence rate of total infection. Given the earlier result, it also means their *number* (or *proportion* since network size is fixed here) is statistically significant.

We repeated the nested ANOVA analysis using simulation results from nine additional networks of size 274 chosen at random, to make ten in total. For all ten networks the spontaneously clearing group was a significant effect in determining the incidence rate of total infection (i.e., 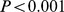 for all ten). On the other hand, when the number of spontaneously clearing nodes in the network was either 64 or 65, the spontaneously clearing group was not a significant effect on the incidence rate of total infection at the 5% level in nine of the ten networks (i.e, 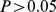 for nine, 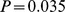 for one). This is consistent with the conclusions from the first network.

### Treatment

**Figure 4:**
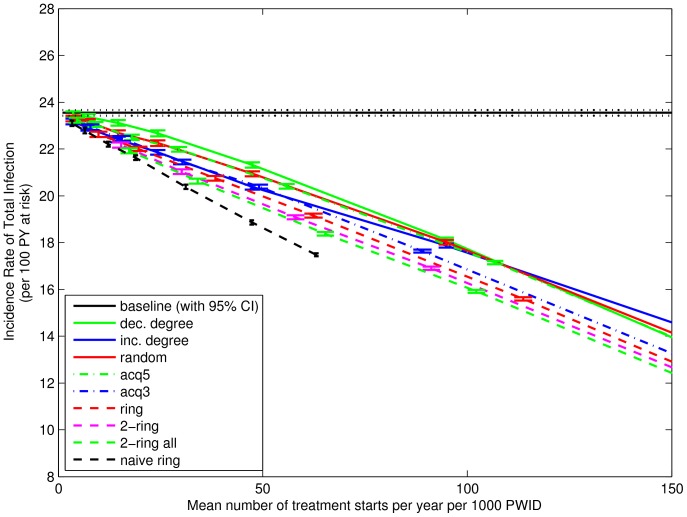
Incidence Rate of Total Infection for Weeks 131–156. Vertical coordinate shows the mean incidence rate of total infection in weeks 131–156, calculated as the mean incidence rates across 500 simulations and then the mean (with 95% confidence interval) across 100 networks. Horizontal coordinate shows the mean number of treatments started in weeks 1–156, calculated as the means across 500 simulations per network, then the mean across 100 networks, and then the mean across 3 years. Strategies that choose nodes at random and ignore the infection status of some (“acq5”) or all (“dec. degree”, “random”) primary contacts have the largest incidence rate of infection. Conversely, the 2-ring strategies and “naive ring” have the lowest incidence rate of infection. Mean treatment starts for “naive ring” are smaller because there are limited numbers of infected nodes available for treatment around randomly chosen uninfected nodes.

Figure 4 shows the effect of the treatment strategies on incidence rate of total infection. Results reported here are for weeks 131 to 156 (where week 1 is the first week beyond burn-in and also the first possible week of treatment.) The vertical axis shows the rate per 100 person-years at risk, and is calculated as the means across 500 simulations per network, then the mean (with 95% confidence intervals) across 100 networks. The horizontal axis shows the average number of treatment initiations started in weeks 1–156. It is calculated as the means across 500 simulations per network, then the mean across 100 networks, and then the mean across 156 weeks. It is reported as the number per year per 1000 PWIDs. For coordinates in the horizontal direction, 95% confidence intervals are smaller than +/−1 (not shown). The incidence rate of total infection with 95% confidence interval for the baseline simulations (“baseline (with 95% CI)”) is shown for comparison. Mean treatment starts for “naive ring” are smaller because there are limited numbers of infected nodes available for treatment around randomly chosen uninfected nodes. With the exception of “naive ring” (which starts from never-infected nodes, unlike the other strategies), for a fixed number of treatment initiations below about 10% per year there is a clear order to the strategies. For all but “inc. degree”, as the average number of people commencing treatment increases, the incidence rate is reduced. In particular, “dec. degree” (often viewed as the best strategy for vaccination) is shown to be the least effective for treatment. Finally, “naive ring”, starting from never infected nodes, appears most effective at reducing the total rate of infection.

The use of the network strategies can be seen as a way of reducing the number of treatments to achieve a desired effect. For example, the effect from treating 47 randomly chosen infected people per 1000 PWID (i.e., 13 in a network of 274) is approximately the same as treating 35 infected people per 1000 using the ring strategy. This difference increases as the treatment frequency increases.

**Figure 5:**
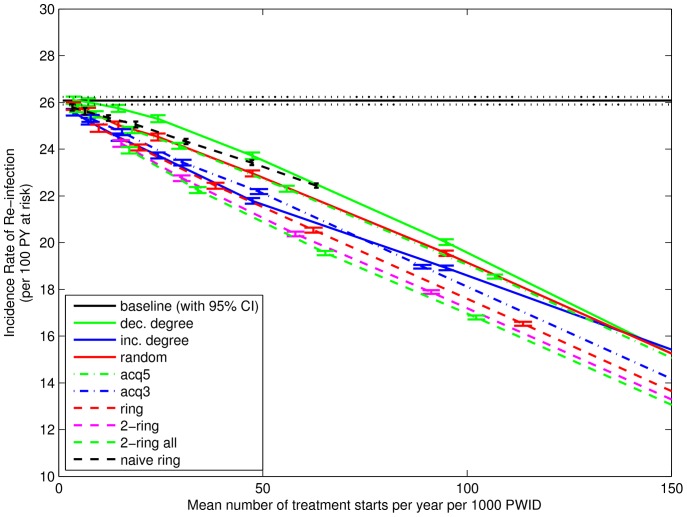
Incidence Rate of Re-infection for Weeks 131–156. Vertical coordinate shows the mean incidence rate of re-infection infection in weeks 131–156, calculated as the mean incidence rates across 500 simulations and then the mean (with 95% confidence interval) across 100 networks. Horizontal coordinate shows the mean number of treatments started in weeks 1–156, calculated as the means across 500 simulations per network, then the mean across 100 networks, and then the mean across 3 years. Strategies that choose nodes at random and ignore the infection status of some (“acq5”) or all (“dec. degree”, “random”) primary contacts have the largest incidence rate of infection. Conversely, the 2-ring strategies have the lowest incidence rate of infection. Mean treatment starts for “naive ring” are smaller because there are limited numbers of infected nodes available for treatment around randomly chosen uninfected nodes.

**Figure 6:**
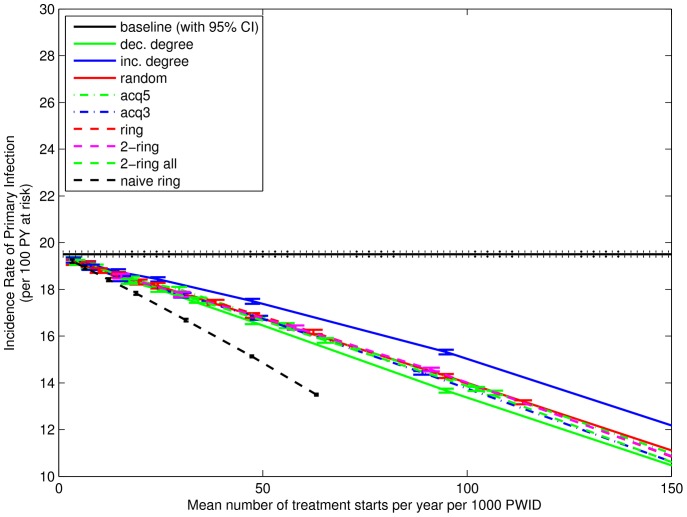
Incidence Rate of Primary Infection for Weeks 131–156. Vertical coordinate shows the mean incidence rate of primary infection in weeks 131–156, calculated as the mean incidence rates across 500 simulations and then the mean (with 95% confidence interval) across 100 networks. Horizontal coordinate shows the mean number of treatments started in weeks 1–156, calculated as the means across 500 simulations per network, then the mean across 100 networks, and then the mean across 3 years. Differences between strategies are smaller than for the incidence rate of total infection and re-infection. The “naive ring” strategy, which treats the primary contacts of randomly-chosen never infected nodes (if they exist) is quite effective. Mean treatment starts for “naive ring” are smaller because there are limited numbers of infected nodes available for treatment around randomly chosen uninfected nodes.

Figures 5 and 6 show comparable results for incidence rate of re-infection and primary infection, respectively, for the various treatment strategies and baseline simulations in weeks 131 to 156. At least four observations can be drawn. Firstly, incidence rates for re-infection are noticeably higher than for primary infection, demonstrating an effect seen in practice in this population [34]. This effect was also observed from simulations in [36] in the context of spontaneously clearing nodes, where it was explained as a “boomerang” effect whereby A infects B, A clears spontaneously, then B re-infects A. The same explanation would apply if A clears by treatment, since in our simulations neither spontaneous clearance nor successful treatment convey any acquired immunity.

**Figure 7:**
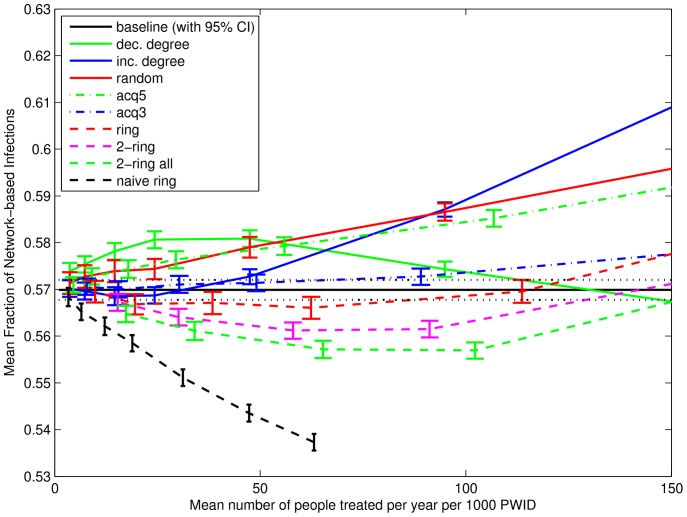
Mean proportion of infections that are network-based. Vertical coordinate shows the mean proportion of new infections in weeks 131–156 that are network-based (i.e., not imported), calculated as the mean proportions across 500 simulations and then the mean (with 95% confidence interval) across 100 networks. Horizontal coordinate shows the mean number of treatments started in weeks 1–156, calculated as the means across 500 simulations per network, then the mean across 100 networks, and then the mean across 3 years. Strategies that choose high-risk nodes (i.e., more primary contacts) at random while ignoring the infection status of some (“acq5”) or all (“dec. degree”, “random”) primary contacts show a larger fraction of network-based infections. At higher treatment frequencies, “inc. degree” shows an increasing fraction of network-based infections as higher-risk nodes are treated. The “naive ring” strategy, which treats the primary contacts of randomly-chosen never infected nodes (if they exist), effectively reduces network-based transmission.

Secondly, except for “naive ring”, an ordering of the strategies using the incidence rate of re-infection is the same as one using the incidence rate of total infection. (Recall that “naive ring” is specifically designed to protect never-infected individuals from infection by treating their contacts.) To better understand the effect of treating nodes but not their infected contacts, and to distinguish the effect of network transmission from the effect of imported infections, Figure 7 shows the average proportion of infections that are network-based (i.e., not imported). The vertical axis shows the proportion of infections in weeks 131 to 156 that are network-based, calculated as the means over 500 simulations per network, then the mean (with 95% confidence interval) over 100 networks. Recall that the number of imported infections in any week depends on the number of susceptibles and the incidence rate of imported infection through equation (1), while the number of network-based infections depends on the number of susceptibles and the number of infected nodes in each susceptible node’s primary contacts. For similar prevalences a higher proportion of network-based infections is a clear sign that a strategy is less effective in reducing transmissions from primary contacts. Unsurprisingly, the strategies that choose nodes at random and ignore the infection status of some (“acq5”) or all (“dec. degree”, “random”) primary contacts see the largest increase in the role of network-based infections. Also notable is the “inc. degree” strategy. At small treatment frequencies, the treated nodes have few contacts and so are at low risk of re-infection. As treatment frequency increases the collection of egos getting treatment grows, and the egos in those collections have increasing numbers of contacts. With more primary contacts comes increased risk of re-infection. The results for “naive ring”, on the other hand, show a comparably larger decrease in the proportion of network-based infections. This is another clear sign that “naive ring” is effectively reducing infections attributable to network transmission.

Thirdly, with the exception of “naive ring”, the differences in the rate of primary infection between the strategies are negligible. For “naive ring”, a trade-off is at work. By focussing on never infected nodes, the incidence rate of primary infections can be lowered, but at the expense of a higher incidence rate of re-infection for other nodes. Whether there is a net benefit from this trade-off is a different matter, but Figure 4 suggests there is.

Finally, we note that the additional benefit from “ring” to “2-ring” is small. In practice, the benefit from using a 2-ring strategy may be outweighed by the additional complexity of finding and treating secondary contacts. Cost-benefit analysis comparing these strategies is left for future work.

**Figure 8:**
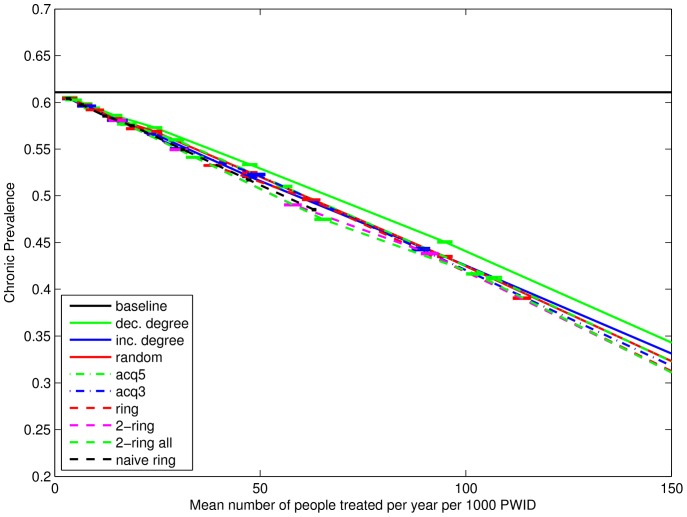
Chronic prevalence at week 156. Vertical coordinate shows the mean chronic prevalence (defined as the proportion of nodes infected constantly for the last 26 weeks, calculated as the mean proportions across 500 simulations and then the mean (with 95% confidence interval) across 100 networks. Horizontal coordinate shows the mean number of treatments started in weeks 1–156, calculated as the means across 500 simulations per network, then the mean across 100 networks, and then the mean across 3 years.

Figure 8 shows similar results for the chronic prevalence at week 156 (defined as the proportion of nodes that have been infected constantly for the last 26 weeks). (Results for prevalence are similar and not shown for brevity.) Baseline chronic prevalence is 61.0%. (It rises above the calibration value of 56% in the three years after burn-in.) Differences between the strategies are small, but the same ordering is apparent, in which “dec. degree”, “acq5” and “random” have the smallest impact and both the “naive ring” and the 2-ring strategies have the largest impact. The relative prevalence reduction is approximately 3.1% and 5.9% for 10 and 20 treatments per year per 1000 PWID at week 156. Also apparent is that the differences between strategies are negligible for treatment frequencies below about 20 per 1000.

### Sensitivity Analysis

We conducted a number of additional analyses to assess the sensitivity of our results to various assumptions. Since we use a static network model, we assessed the sensitivity of our treatment results to the choice of the particular weeks after burn-in used for reporting results. Specifically, we limit the time period of interest to the first 52 weeks following burn-in. On this shorter period the assumption of a static network is more realistic. To do this we calculate the incidence rate of total infection for each of the nine strategies on weeks 27 to 52 which provides 26 weeks for the treatments to produce an effect. We calculate the number of treatments on weeks 1 to 52. Results for the treatment strategies are qualitatively similar. That is, a ranking of the strategies from most to least effective is the same. The main difference is that the size of the impacts were not as great, due to a smaller period for treatment to have an effect. This is not shown for brevity. Importantly, this shows that even over a shorter time period in which the assumption of a static network is more realistic, our conclusions ranking the various treatment strategies do not change.

We conducted additional simulations to account for uncertainty in input parameters to our model. In total, 14 additional scenarios were investigated under five treatment strategies (decreasing degree, random, ring, 2-ring, naive ring). These are described in Supporting Information S1. With the exception of a scenario in which 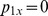 so all infections are from the importing source, our results consistently show that incidence rates of total infection under treatment can be ranked in the following order: decreasing degree>random>ring>2-ring>naive ring.

Finally, to investigate the suitability of our assumption of a static network, we performed additional analysis on the duration of edges in our empirical network [36]. As part of the Melbourne study, respondents reported on the time since first using and last using with each nominee. Of the 263 edges in our empirical network, we have such data for 250 edges. In the case of multiple responses per edge by the same respondent, the first was used. In the case both respondents reported these durations, those with the smaller network identifier were used (an arbitrary choice). For this group, 104 (i.e., 41.6%) report first using with the nominee at least three years ago. But this ignores right censoring, which occurs if there is still activity between members of the dyad. We say a dyad’s duration is right-censored if the last activity was reported to be at most 75 days ago. Then, 11 durations are not censored (time since last use: median 91, range 91–1095), 239 are censored, and from the Kaplan-Meier estimator, 97.0% (95% CI: 94.6% –99.4%) of these edges have duration at least three years. The 75 day cutoff is conservative since it is less than the period between interviews. Larger cutoffs increase the estimated percentage of dyads with duration at least three years. These estimates do not account for any possible bias from the network-based sample design.

## Discussion

Our results demonstrate the PWID network plays an important role in hepatitis C transmission through both the number of contacts and the attributes of one’s sharing partners. Understanding the PWID network is likely to play an important role in the effective and efficient roll out of HCV treatment of PWID over the next 20 years. In this study, strategies that include treatment of both primary and secondary contacts are the most effective in reducing incidence rates of re-infection and total infection, for similar numbers of treatment starts.

We have shown that the number of network partners plays an important, direct role in determining the time to primary infection. The time to primary infection for someone with six contacts may be less than half that of someone with one contact. Our network model also suggests location, age and frequency of injecting contribute to the configuration of the network, thus playing an indirect role in risk of infection too. We have also shown that the difference in time to primary infection between “less-frequent” and “more-frequent” injector is roughly the same as having one additional network contact. Thus, it may be more effective for health promotion campaigns to focus on the social context in which risk behaviours take place (e.g., with whom, with how many different people), rather than simply focusing on the behaviours themselves (e.g., sharing injecting equipment).

In the context of treatment, treating an individual without treating their contacts leaves a reservoir of virus as a source of re-infection (in the absence of acquired immunity) and so those treated are at high risk of re-infection. Treatment strategies that take advantage of the contact network of PWID are more effective in lowering both the incidence rates of re-infection and total infection. For similar numbers of treatment starts above about 20 per year per 1000 PWID, the most effective strategies at lowering incidence rates of re-infection in this study treat infected primary and secondary contacts of infected PWID as well (i.e., “2-ring”, “2-ring all”). The strategy treating primary contacts but not secondary contacts (“ring”) was almost as effective. The least effective strategies treat infected PWID selected at random (“random”), or chosen by decreasing numbers of primary contacts (“dec. degree”). The lack of effectiveness of “dec. degree” as a treatment strategy is in stark contrast to the widespread belief that targeted vaccination is the most effective *vaccination* strategy. The possibility of re-infection appears to play an important role in our results. But our networks lack hubs. An interesting question for future work is whether the “dec. degree” strategy is relatively more effective for networks with hubs.

A common way to think of an infectious disease spreading is to imagine the disease spreading away from an index case at the start (e.g. SARS, influenza) or end (e.g. smallpox eradication) of an outbreak. In the context of HCV in Melbourne, Australia, where half or more of the population of interest (PWIDs) are already infected, it may more more helpful to think of infection transmitted *into* uninfected people. Thus we also studied a strategy (“naive ring”) that treats infected primary contacts of *uninfected* PWID as a means of protecting their uninfected status. Although not clinically practical (clinicians will not normally have contact with uninfected PWID and their close contacts) it serves to demonstrate what is possible with a network strategy. It was by far the most effective strategy at reducing the incidence rate of primary infection and subsequently the incidence rate of total infection too.

We have demonstrated a reduction in chronic prevalence through treatment. Martin et al. ([13], Figure 6) reported larger relative prevalence reductions of about 6.7% and 13% over a longer five year period for an 80% effective treatment, which are roughly similar results considering we report over a three year period. Our results also show a similar ranking to results for incidence rates, in which decreasing degree shows the smallest effect, the 2-ring strategies show the largest effect, and the random strategy is somewhere in between. However, with the exception of one strategy (“dec. degree”) the differences between the strategies are small. This is a consequence of the limited time period under consideration. Recall that even a difference of two people infected in a network of size 274 is less than 1% difference in prevalence. For the differences to appear large requires more time for the strategies to have an impact. So, here we can show the relative impact of the strategies on chronic prevalence, but a dynamic network simulating a longer period is really needed to assess the size of the differences on the time scale of a long-term public health intervention.

In the context of HCV transmission we have shown that the number (and proportion) of spontaneously clearing nodes has a statistically significant effect on the network-wide incidence rate of total infection. On the other hand, for a fixed number of such nodes, their arrangement within the network does not have a statistically significant effect on incidence rate of total infection. This suggests that apart from their risk of re-infection, the effect of spontaneously clearing nodes is a local effect [36] in which they are a lower risk as a source of infection to their primary contacts.

Our work is novel for a number of reasons. 1) Our study investigates an anticipated HCV treatment, rather than preventative vaccines. 2) Unlike other network-based intervention studies we do not consider the beginning or the end of an epidemic. Rather, HCV is essentially endemic, infecting about half the network. 3) We directly compare a number of network-based interventions in this population, including ring vaccination with secondary contacts. 4) The contact network model of PWID is empirically grounded [37] and demonstrated to capture a number of features of an empirical contact network. The model uses recently developed statistical approaches in social network analysis to include features previously demonstrated to be relevant to human interaction, such as clustering, attribute-based homophily and social circuit dependence. Indeed, it is the first PWID network model to explicitly model social circuit dependence. Moreover, our network model is not scale-free. 5) The individual-based transmission model [36] includes nodes that can spontaneously clear and be re-infected, and transmission of infection from sources other than network neighbours, at a rate estimated from empirical data. These “imported infections” recognise both the limitations of including all network partners in empirical studies and of using a static network to model time intervals longer than those used to define a contact.

This study has several limitations. We have modelled a three year period following burn-in using a static network, which we recognise is an approximation. As described in Welch et al. [28], a static network is a natural place to begin research. There is also strong evidence that the empirical network used as the basis for our contact network model has a large proportion of injecting relationships that have persisted over the last three years. This should not be taken to mean that activity along each dyad occurs consistently. It was previously estimated that activity along each edge occurred in about 19% of weeks [36]. How this activity clusters in time is an interesting issue for a dynamic model. Nevertheless, the simulations of various treatment strategies show qualitatively similar results over the first year, so the use of a three year period is not crucial to our general conclusions on treatment. Combining HCV transmission with an empirically grounded dynamic network model is an interesting direction for future work. Work on this is already under way.

We deliberately considered the use of a treatment rather than a vaccine because this is a major issue with the considerable advances in direct-acting antiviral agents, and there is currently no vaccine for HCV. Necessarily, treatment is targeted at sero-positive PWID. This differs from the results in Hahn et al. [21] which considered a prophylactic vaccine. A key difference, of course, is the latter is also given to HCV-naive individuals which can provide a greater opportunity to lower primary infection rates. In addition, those antiviral treatment regimens are expected to have substantially better tolerability and it should therefore become possible to treat individuals and their close contacts simultaneously.

We have not explicitly modelled the arrival of new injectors to the network. This means our results on the time to primary infection and “naive ring” treatment strategy assume the contacts of new injectors are similar to others in the network. Our results on “naive ring” in particular highlight the need for a dynamic network model as future work, with special emphasis on new members to the drug-injecting scene. Those people represent a pool of uninfected people. How they form contacts early in their injecting careers must play a key role in both their risk of primary infection and strategies to prevent primary infection. Such a model would also give an indication of the role of population turnover in the infecting scene as newer, never-infected people enter the injecting scene while more experienced, infected people leave.

The treatment strategies considered here do not explicitly target recent infections or new PWID. As a result differences in the rate of primary infection between the strategies are negligible (with the exception of the “naive ring” strategy). We leave study of such strategies for future work.

We have assumed the probability of infection is constant throughout the duration of an infection. Currently there is no consensus on the variability in infectivity following infection, and we feel any other choice would be arbitrary in the absence of supporting data. We think the role of increased infectivity in the first acute phase of infection would be minor over short durations when over 50% of nodes in the network have already been infected by the end of burn-in. We also suspect increased infectivity in the acute phase will be more important for a dynamic model where the arrival and early days of HCV-uninfected people in the network are explicitly modelled.

Our transmission model assumes no acquired immunity. Under this assumption, “boomerang” infections, in which A infects B, A becomes uninfected, then B infects A, can play an important role in re-infection. We feel this is a conservative assumption in the context of a model of the effects of treatment on HCV incidence and prevalence. Results from empirical studies of HCV re-infection following spontaneous clearance of prior HCV infection have been variable, with some reporting much lower rates of re-infection compared to primary infection [65]–[69], and others reporting rates of re-infection equal to or higher than the rates of primary infection [34], [70]–[73]. Recently, it has been recognised that much of this variation can be attributed to variation in HCV testing intervals between studies, where studies with lengthy test intervals miss spontaneously clearing re-infection that occurs between HCV tests and therefore underestimate the re-infection rate [74]. Whilst most empirical studies of HCV re-infection following successful antiviral treatment have found low rates of re-infection [10], [11], [65], [67], [75], [76], studies of HCV re-infection following successful antiviral treatment in PWID in prison and HIV-infected men who have sex with men have found high rates of re-infection [12], [77], [78]. With the advent of new highly-effective and increasingly tolerable treatment regimens, the characteristics of the people receiving treatment may change and re-infection rates following successful treatment will need to be closely monitored. If later clinical results establish that spontaneous clearance or successful treatment leads to acquired immunity, our model will overestimate the rate of re-infection and the relative advantages of the various intervention strategies would change.

The imported infections included in the transmission model provide a way to model risk of infection from sources other than primary contacts. It is a modelling device that reflects limitations in modelling the contact network, which in turn reflects difficulties with collecting data on this difficult-to-reach population of individuals. Since this risk of infection is independent of the contact network and lacks heterogeneity (except for the difference in incidence rate between less-frequent and more-frequent injectors), our results should be conservative with respect to differences between network-based treatment strategies.

Our investigation of contact referral strategies like ring treatment assumes all infected contacts are treated. In reality, only a fraction of those contacts would be treated. For example, some contacts may be unwilling to have their HCV status determined, while others may reject treatment despite being infected. Although we have not explicitly modelled these effects, a number of aspects of our simulation mitigate these differences. We assume the treatments are only effective in 80% of people, so incomplete elimination of infection in primary contacts is already included. Futher, we include importing of infection which means a node continues to have risk even if all contacts are uninfected. Finally, the acquaintance immunisation strategies “acq3” and “acq5” treat only a fraction of a node’s primary contacts, thus giving a sense of the difference incomplete treatment of primary contacts can make (albeit when untreated primary contacts are not randomly chosen, but chosen by node degree.).

It would be interesting to do a direct comparison of our network based HCV model treating random nodes, for example, with a deterministic mixing model using similar treatments and similar treatment numbers. This would help aid interpretation of results from mixing models. This is left for future work.

## Supporting Information

Supporting Information S1 (DOC)
