# Supplementary material for: Hepatitis C Transmission and Treatment in Contact Networks of People Who Inject Drugs
Source: PLoS One. 2013 Nov 1;8(11):e78286. doi: 10.1371/journal.pone.0078286 (PMC3815209; doi:10.1371/journal.pone.0078286)
Supplement: Supporting Information S1 — (DOC) [file pone.0078286.s001.doc]

**Supporting Information Text S1: Sensitivity Analysis**

Model parameters

To account for uncertainty in the input parameters of our model, we examined the sensitivity of our treatment intervention results to 7 key model parameters. We examined two alternative values for each parameter. With the exception of one parameter (the time from treatment epoch to the end of infectivity), the values are chosen such that one is significantly larger and the other significantly smaller than the value used for the standard baseline. Table S1.1 describes each scenario. For the first 5 pairs listed, the high and low parameters values correspond to endpoints of 95% confidence intervals. (In fact, the upper limit for the incidence rate of imported infections should be 20.6, which is higher than the standard value of 18.9 for the network wide incidence rate of total infection. Thus, we use the latter.) For the matrix of weekly sharing probabilities for low and high frequency PWID, we take “low” and “high” to mean less difference and more difference, respectively, between the two groups. Since any constant multiplier of the matrix is absorbed into the value of $p_{1x}$ at calibration, this degree of difference is the key feature. For the time from treatment epoch to the end of infectivity, since its value was conservatively chosen, both alternative scenarios for that parameter use values smaller than for the standard model.

| **Table S1.1** Sensitivity Analysis Scenarios | | |
| --- | --- | --- |
| **Parameter** | **Baseline value** | **Alternate Values** |
| *Parameters set at Calibration* | | |
| Incidence rate of total infection | 18.9 | 13.7, 25.4 |
| HCV prevalence at end of burn in | 0.56 | 0.51, 0.62 |
| *Transmission Model Parameters* | | |
| Proportion of spontaneous clearance nodes | 0.25 | 0.15, 0.35 |
| Incidence rate of imported infections (at 56% prevalence) | 9.0 | 2.9, 18.9 |
| Mean time to acute spontaneous clearance | 7 | 5, 9 |
| Weekly sharing probabilities for low and high frequency PWID | $\left(\begin{matrix} 0.19 & 0.18 \\ 0.18 & 0.24 \end{matrix}\right)$ | $\left(\begin{matrix} 0.2 & 0.2 \\ 0.2 & 0.2 \end{matrix}\right)$, $\left(\begin{matrix} 0.14 & 0.18 \\ 0.18 & 0.28 \end{matrix}\right)$ |
| *Treatment Parameters* | | |
| Weeks from treatment epoch to end of infectivity | 10 | 2, 6 |

For each scenario, the following procedure was carried out:

1. Calibration, as described in Rolls et al. (2012), was performed against the empirical network to obtain the value of $p_{1x}$, the probability of transmission from a single sharing event, to achieve the target incidence rate of total infection (18.9 unless the parameter under investigation) at the target HCV prevalence (56% unless the parameter under investigation).
2. For each of the 100 graphs considered, the duration of the burn in to achieve the target prevalence at the end of burn in was determined.

To reduce the computational load arising from these 14 scenarios, it was decided to consider a subset of the treatment strategies (decreasing degree, random, ring, 2-ring, naive ring), restrict interest to treatment frequencies in the range 20—30 per year per 1000 PWID, and simulate 100 (not 500) times per network.

We present results for a treatment frequency of 25 per year per 1000 PWID, although results in the range 20—30 are similar. Since the number of treatments varies randomly for the network-based strategies and none of the frequencies for “decreasing degree” and “random” are exactly 25 per year per 1000 PWID we use the following approach.

For each treatment strategy$s$ and each network $k$we performed the following analysis:

1. Compute the incidence rate of total infection for weeks 131 to 156,$R_{ij}$, and the mean number of treatments (as number per year 1000 PWID), $m_{ij}$, where $i=1,2,3$ denotes one of three treatment epoch intervals simulated, and $j=1,2,\ldots,100$ denotes the particular simulation.
2. Fit a linear regression model with $m_{ij}$as the independent variable and $R_{ij}$ as the dependent variable using the 300 points$\{(m_{ij},R_{ij}), i=1,2,3, j=1,2,\ldots,100\}$.
3. Use the regression model to find the incidence rate of total infection at 25 per year per 1000 PWID,$Y_{sk}$, by interpolation. (Results reported here fit a quadratic curve model for the interpolation. Interpolated values from fitting a line are almost identical and omitted for brevity.)

Then, to determine whether strategy $s_{1}$ leads to smaller incidence rates of total infection than strategy$s_{2}$, we use a binomial test. That is, we count the number of cases $X$ (out of 100) where$Y_{s_{1}k}<Y_{s_{2}k}$. Then $X$ has a binomial distribution with parameters $n=100$ and $p=1/2.$

For a statistical test with the null hypothesis that incidence rates for strategy $s_{1}$are equal to $s_{2}$and alternative hypothesis that incidence rates for strategy $s_{1}$ are smaller than for strategy$s_{2}$, we check whether $X\geq59,$ the critical value for a test at 5% significance. We use this test because it is sensitive to the direction of inequalities but not the size of the differences, and there is a concern that the variances of incidence rates may differ between graphs making other tests inappropriate.

Table S1.2 summarises values of$p_{1x}$, incidence rates of total infection at baseline. Columns 5—9 show incidence rates of total infection at 25 treatment initiations per year per 1000 PWID for each of the five strategies considered, and the rank of each strategy, where (1) denotes the smallest (most desirable) incidence rate and (5) denotes the largest. Incidence rates are shown in boldface if they are significantly smaller than baseline in the binomial test described above. Rank $(i)$, $i=1,2,3,4,$ is shown in boldface if incidence rates are significantly smaller than for rank $(i+1)$ in the binomial test described above.

With the exception one scenario (importing rate 18.9), and occasional random variation, these results consistently demonstrate results from our standard model on incidence rates of total infection under these strategies:

Decreasing degree > Random > Ring > 2-Ring > Naive ring.

In terms of the size of the *effect* on incidence rates, the inequalities would be reversed:

Decreasing degree < Random < Ring < 2-Ring < Naive ring.

For the scenario with importing rate 18.9 and$p_{1x}=0$, there is no transmission within the modelled network so we would not expect one strategy to perform better than the other. Indeed, with the exception of rank (2) (for which$X=60$, just barely larger than the critical value), none of the ranks is statistically significant.

| **Table S1.2** Effect of Treatment Strategies on Incidence Rate of Total Infection | | | | | | | | |
| --- | --- | --- | --- | --- | --- | --- | --- | --- |
| **Parameter varied** | **Parameter value** | $p_{1x}$ | **Incidence rate at baseline** | **Mean Incidence Rate at 25 Treatments per Year per 1000 PWID (strategy rank)** | | | | |
|  |  |  |  | **Dec. degree** | **Random** | **Ring** | **2-Ring** | **Naive ring** |
| Standard | n/a | n/a | 23.5 | **22.6** (5) | **22.2 (4)** | **21.6 (3)** | **21.3 (2)** | **20.8 (1)** |
| Incidence rate of total infection | 13.7 | 0.00386 | 15.1 | **14.1** (5) | **14.0** (4) | **13.7 (3)** | **13.5 (2)** | **13.4 (1)** |
|  | 25.4 | 0.01764 | 33.3 | **32.8** (5) | **31.8 (4)** | **30.8 (3)** | **30.3 (2)** | **29.3 (1)** |
| HCV prevalence at end of burn in | 0.51 | 0.01213 | 23.3 | **22.3** (5) | **22.0 (4)** | **21.2 (3)** | **20.9 (2)** | **20.2 (1)** |
|  | 0.62 | 0.00728 | 22.6 | **21.9** (5) | **21.3 (4)** | **20.7 (3)** | **20.7** (2) | **20.4 (1)** |
| Proportion of spontaneous clearance nodes | 0.15 | 0.01011 | 24.4 | **24.1** (5) | **23.5 (4)** | **22.5 (3)** | **22.2 (2)** | **21.5 (1)** |
|  | 0.35 | 0.00942 | 21.7 | **20.5** (5) | **20.4 (4)** | **19.8 (3)** | **19.5 (2)** | **19.3** (1) |
| Incidence rate of imported infections (at 56% prevalence) | 2.9 | 0.02216 | 26.8 | 27.1 (5) | **26.1 (4)** | **24.8 (3)** | **24.4 (2)** | **23.4 (1)** |
|  | 18.9 | 0.0 | 22.9 | **21.3** (4) | **21.2** (5) | **21.0** (3) | **20.8 (2)** | **20.9** (1) |
| Mean time to acute spontaneous clearance | 5 | 0.00998 | 23.1 | **22.4** (5) | **22.0 (4)** | **21.3 (3)** | **21.0 (2)** | **20.4 (1)** |
|  | 9 | 0.01041 | 23.4 | **22.6** (5) | **22.1 (4)** | **21.4 (3)** | **21.1 (2)** | **20.5 (1)** |
| Weekly sharing probabilities for low and high frequency PWID | $\left(\begin{matrix} 0.2 & 0.2 \\ 0.2 & 0.2 \end{matrix}\right)$ | 0.01062 | 23.9 | **23.3** (5) | **22.7 (4)** | **22.0 (3)** | **21.7 (2)** | **21.2 (1)** |
|  | $\left(\begin{matrix} 0.14 & 0.18 \\ 0.18 & 0.28 \end{matrix}\right)$ | 0.01051 | 18.0 | **17.3** (5) | **17.1 (4)** | **16.5 (3)** | **16.2 (2)** | **15.9 (1)** |
| Weeks from treatment epoch to end of infectivity | 2 | 0.00995 | 23.5 | **22.4** (5) | **22.1 (4)** | **21.5 (3)** | **21.3 (2)** | **20.6 (1)** |
|  | 6 | 0.00995 | 23.5 | **22.7** (5) | **22.3 (4)** | **21.6 (3)** | **21.3 (2)** | **20.7 (1)** |
